# LC–MS based case-by-case analysis of the impact of acidic and basic charge variants of bevacizumab on stability and biological activity

**DOI:** 10.1038/s41598-020-79541-2

**Published:** 2021-01-29

**Authors:** Sumit Kumar Singh, Deepak Kumar, Himanshu Malani, Anurag S. Rathore

**Affiliations:** Department of Chemical Engineering, Indian Institute of Technology, Hauz Khas, New Delhi, 110016 India

**Keywords:** Biotechnology, Health care

## Abstract

The present study investigates the impact of charge variants on bevacizumab's structure, stability, and biological activity. Five basic and one acidic charge variants were separated using semi-preparative cation exchange chromatography using linear pH gradient elution with purity > 85%. Based on the commercial biosimilar product's composition, two basic variants, one acidic and the main bevacizumab product, were chosen for further investigation. Intact mass analysis and tryptic peptide mapping established the basic variants' identity as those originating from an incomplete clipping of either one or both C-terminal lysine residues in the heavy chain of bevacizumab. Based on peptide mapping data, the acidic variant formation was attributed to deamidation of asparagine residue (N84), oxidation of M258, and preservation of C-terminal lysine residue, located on the heavy chain of bevacizumab. None of the observed charge heterogeneities in bevacizumab were due to differences in glycosylation among the variants. The basic (lysine) variants exhibited similar structural, functional, and stability profiles as the bevacizumab main product. But it was also noted that both the variants did not improve bevacizumab's therapeutic utility when pooled in different proportions with the main product. The acidic variant was found to have an equivalent secondary structure with subtle differences in the tertiary structure. The conformational difference also translated into a ~ 62% decrease in biological activity. Based on these data, it can be concluded that different charge variants behave differently with respect to their structure and bioactivity. Hence, biopharmaceutical manufacturers need to incorporate this understanding into their process and product development guidelines to maintain consistency in product quality.

## Introduction

The rapidly increasing global burden of cancer has lately become a topic of significant concern. This is evident from the 2015 report by the American Cancer Society, which attributes 1 in 8 deaths to cancer globally^1^. Amongst the various cancer forms, lung cancer is the second most commonly diagnosed form of cancer^2^. Bevacizumab, also known as Avastin, is a popular monoclonal antibody-based drug used widely to treat various cancers, including lung cancer, cervical, colorectal, and renal carcinomas^3^. Bevacizumab targets the vascular endothelial growth factor (VEGF), which functions as the source of nourishment to the cancer cells by allowing blood vessels growth via a process known as angiogenesis^4^.

Monoclonal antibodies are generally purified from mammalian cell culture using a platform process comprising affinity, hydrophobic interaction, ion exchange column chromatography with intermittent membrane ultrafiltration, and diafiltration steps^5^. However, the final purified product is often a cocktail of various product variants a nd process-related impurities that could reduce the drug's therapeutic efficacy and, worse, pose safety challenges^6^. Charge heterogeneity involves product variants with altered charge characteristics and may vary in their clinical impact, vis-à-vis the main product^7^. It is known that nearly the entire production process affects the charge heterogeneity of the final product^8^, and as a result, manufacturers spent considerable resources designing a process that can manufacture the product of defined charge heterogeneity^9^.

The issue is of particular concern for the development of biosimilars^10^ as the regulatory expectation is that the biosimilar manufacturer will meet the charge variant profile of the innovator product^11,12^. However, it has been pointed out that not all acidic variants or basic variants are similar in how they impact product safety and efficacy^13^. Hence, there is a need to understand the impact of the individual charge variants and this can be achieved by isolating the individual variants, and assessing the structural changes and their impact due to underlying modification^14^.

Charge variants of mAbs are formed due to protein modifications such as methionine oxidation, lysine clipping, glycation, glycosylation, and disulfide shuffling^15^. Biophysical tools such as circular dichroism, fluorescence spectroscopy, and fourier transform infrared spectroscopy (FTIR) can provide information about the secondary and tertiary structure of a protein^16^. Circular dichroism reflects the changes in the secondary structure of proteins with far-UV scanning in the wavelength region 190–250 nm^17^. The tertiary structure of proteins that exhibit good ellipticity can also be assessed using CD spectroscopy by recording the spectrum in the near-UV wavelength region of 250–350 nm^18^. The stability of proteins as a function of pH, temperature, and other environmental factors can be examined using thermal or chemical denaturation, followed by recording the CD spectrum in far/near UV regions^19,20^. The presence of aromatic amino acids, such as tryptophan, tyrosine, and phenylalanine, renders them suitable for structural characterization using fluorescence spectroscopy^21^. By virtue of higher extinction coefficient, quantum yield, and sensitivity to polar/aqueous environment, tryptophan is an ideal probe for assessing structural perturbations that result from the modifications that contribute to the formation of charge variants^22^. If any, the changes can be inferred from red/blue shifts of the emission maxima, which is around 350 nm when tryptophan residues are exposed to polar solvents and 320 nm when it is buried deep inside the protein core^19^. The melting temperature of proteins can also be determined using fluorescence spectroscopy^23^.

Charge variants of mAbs are typically separated and purified using cation exchange chromatography^24,25^. However, since the properties of the target product (mAb) and the corresponding aggregate are near-similar, as far as the binding to the ion exchange column is concerned, it is nearly impossible to obtain baseline separation of a mAb or a charge variant and the corresponding aggregate species^26^. Therefore, the aggregation level needs to be kept under consideration when performing biophysical characterization of mAb charge variants. Size exclusion chromatography (SEC), CD spectroscopy, fluorescence spectroscopy, and fourier transform infrared spectroscopy (FTIR) are some contemporary biophysical tools researchers have used to monitor aggregation of proteins^27^. Protein aggregation has been correlated with an increase in the β-sheet content, and this can be quantified by using advanced algorithms such as area overlap (AO), second derivative, and spectral correlation coefficient (SCC) from the FTIR spectrum^19,28^.

In the present study, we have isolated the charge variants (5 basic and one acidic variant) of bevacizumab using cation exchange chromatography. These variants were chosen based on the composition of the commercial product. Further, the isolated variants were characterized using biophysical tools, including CD spectroscopy, FTIR, and fluorescence spectroscopy, to monitor these variants' secondary and tertiary structures vis-à-vis the main product.

## Results

### Two basic variants and one acidic variant were common charge variants in commercial biosimilar and in-house bevacizumab product

**Figure 1:**
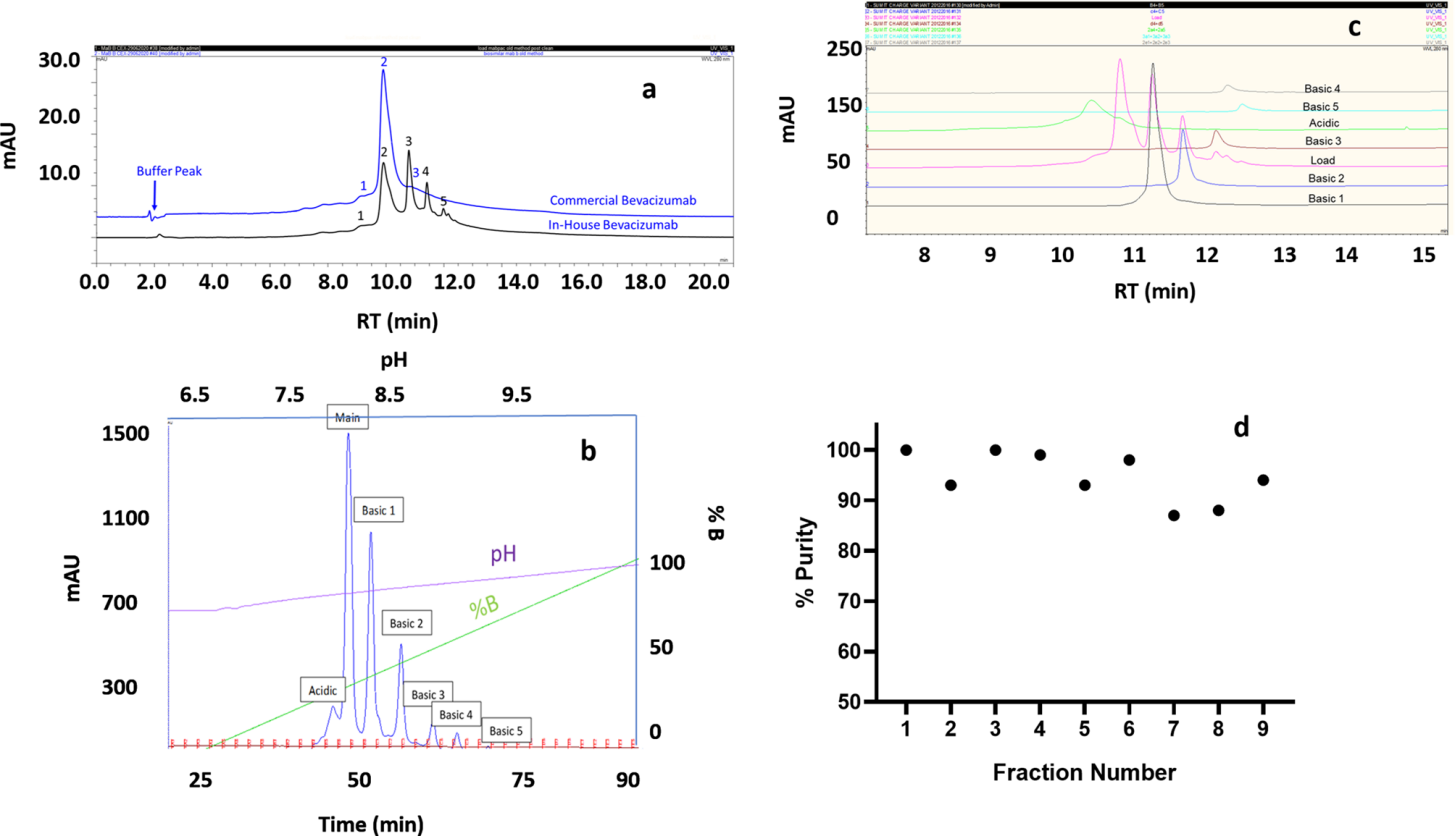
Charge variant separation of bevacizumab using analytical cation exchange chromatography (CEX). (**a**) Comparison of the charge variant profiles of a commercial and an in-house purified bevacizumab product. (**b**) preparative scale separation of charge variants of bevacizumab using pH gradient CEX, showing the separation of five basic variants, and one acidic variant. (**c**) CEX profiles of the isolated individual charge variants. (**d**) Assessing the purity of the isolated charge variants using analytical cation exchange chromatography. The charge variants that were common in the commercial samples and having purity > 85% and aggregate content < 3% were only shortlisted for further evaluation. Basic variant 1, basic variant 2, acidic variant, and bevacizumab main product passed these criteria.

The charge variant separation of the commercial and in-house bevacizumab was performed using CEX employing a pH gradient to assess the distributional differences between them. The purpose was to isolate these variants into two groups: first, including those charge variants present in the commercial biosimilar sample, and the second, the group of variants that are absent in the biosimilar but are present in the in-house sample. As shown in Fig. 1a, the peak with maximum absorbance is the main peak, while the five peaks eluting post main peak are referred to as basic variants. The peak eluting before the main peak has apparent pI lower than the main peak and therefore is referred to as the acidic variant. It is seen that the distribution of the charge variant in the in-house bevacizumab is comprised of five basic variants, one acidic variant, and one unmodified bevacizumab product. In contrast, the commercial product was less heterogeneous in terms of charge variant composition and primarily was composed of one acidic, one basic, and one unmodified bevacizumab product. While the relative % composition of the acidic variant was almost similar in both the products, the basic 1 variant was relatively higher in the in-house product. It is also seen that basic 2 through basic 5 variants were absent in the commercial product (Table 1).

**Table 1:** Listing the distribution of the charge variants of bevacizumab, a commercial biosimilar, and an in-house developed bevacizumab product.

Peak	Relative retention time (RRT)	Relative %	
		In-house Bevacizumab	Commercial Bevacizumab
Acidic	9.6	8.0	6.2
Main	9.9	34.7	77.1
Basic 1	10.8	26.4	16.7
Basic 2	11.4	14.5	0
Basic 3	11.9	6.8	0
Basic 4	12.1	3.1	0
Basic 5	12.3	6.5	0

### Bevacizumab charge variants were isolated with high purity using semi-preparative chromatography

In order to obtain enough samples for each of the charge variants to facilitate their in-depth characterization, CEX separation employing a pH gradient was performed on a semi-preparative scale chromatographic system (Fig. 1b). The purpose was to obtain the resolution of the variants like that was obtained at the analytical scale to obtain material in enough amounts. These objectives were met with the separation method, and fractions of fixed volume (1 ml) were collected throughout the elution window. The collected fractions were pooled such that one sample represented a predominantly single charge variant specie of bevacizumab (> 85% of the species under consideration).

The pooled fractions from the semi-preparative CEX step were analyzed for purity using analytical CEX and the aggregate content using SEC (Fig. 1c). Analysis of the starting material (load) using analytical CEX showed that apart from the main peak (unmodified bevacizumab), five basic variants and an acidic variant were separated. Therefore, the number of variants was in agreement with what was obtained at the preparative scale (Fig. 1c). It is seen in the load material that the first two basic variants dominate in terms of the relative abundance of the overall charge variant content of the bevacizumab (> 80% of the total charge variants). The purity of the individual isolated charge variants was > 85%, as shown in Fig. 1d. It was also found that the later eluting fractions comprised of the aggregated species and a cut-off of 3% aggregates in the individual charge variants were targeted during the pooling of collected fractions. The 3% cut off was made based on the maximum permissible aggregate content in bevacizumab as per the various regulatory submissions^29^. Aggregate content in the fraction containing Basic variants 2, 3, 4, and 5 exceeded this threshold. These variants were also not found in the commercial biosimilar product.

Next, we shortlisted basic 1, basic 2, acidic, and bevacizumab main products for in-depth structural and functional characterization based on their presence in the commercial product. Basic 2 variant, even though absent in the biosimilar, was included for analysis in this study due to a very similar modification (lysine clipping) to the basic 1 variant. Further, specific literature reports show that some of the basic variants (originating from lysine clipping) exhibit enhanced pharmacokinetic half-life and better therapeutic efficacy. We aimed to test this hypothesis for bevacizumab lysine variants by pooling them in different proportions with the main product and assessing their impact on the function.

### LC–MS characterization shows observed charge heterogeneity is not due to glycosylation differences among variants

**Figure 2:**
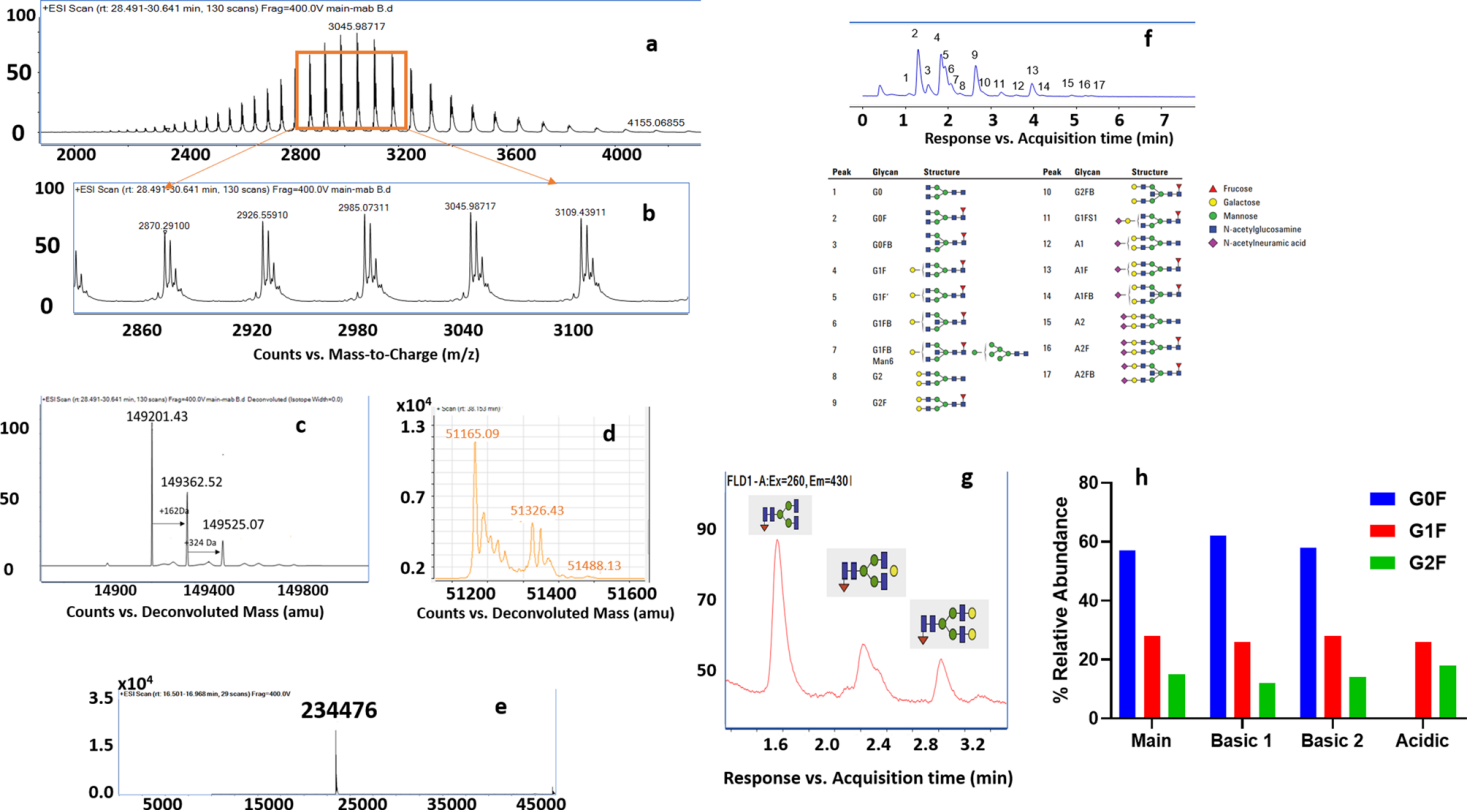
(**a**) Raw MS spectrum of the bevacizumab main product; (**b**) zoomed-in image of the MS spectrum showing the presence of three structural isoforms; (**c**) deconvoluted MS spectra of the bevacizumab main product showing glycan moderate glycan heterogeneity comprising of G0F, G1F and G2F species; (**d**) Deconvoluted MS spectra of the heavy chain of bevacizumab main product showing that glycan occupancy in the product is in the heavy chain; (**e**) Deconvoluted MS spectra of the light chain of the bevacizumab main product showing no glycan occupancy in the light chain; (**f**) 2-AB linked glycan library of 17 human IgG glycan constructed using fluorescence detection with the corresponding annotation of the peaks; (**g**) confirmation of the glycan composition of the bevacizumab main product using released glycan assay; (**h**) comparison of the distributional difference of the glycosylation in the isolated charge variants of bevacizumab.

The intact mass spectrum indicated a moderate heterogeneity in the bevacizumab. This is evident from the three isoforms corresponding to the different glycan forms seen in the zoomed intact spectrum (Fig. 2a,b). The most abundant peak corresponds to a mass of 149,201.43 Da with a mass error of 10 ppm. This represents a difference of 2892.3 Da from the calculated theoretical mass of the aglycosylated form. It is to be noted here that the experimental mass of the unmodified bevacizumab is less than 256 Da of the theoretical mass, indicating that both the C-terminal lysine residues of the heavy chain are clipped. This mass shift correlates with complex biantennary glycan structure with core fucose and the two branches terminating with zero (G0) galactose. Besides, other prominent peaks in the deconvoluted spectrum correspond to masses of 149,362.52 Da (+ 161.09 Da) and 149,525.07 Da (+ 323.64 Da). The mass shift of + 161 Da and + 324 Da represents a variant with the usual glycan core terminating with one (G1) and two (G2) galactose moieties (Fig. 2c). However, the mass shift is offset by ~ 1 Da for both G0F and G1F forms from the theoretical shift of 162 Da for galactose. To confirm the deconvoluted intact mass spectrum's peak annotation, 2-AB labeled released glycan assay with fluorescence detection was performed. Figure 2d shows the library constructed using 2-AB labeled released human IgG glycans, comprising of 17 different glycan species used to support the annotation of the peaks from the released glycans from the bevacizumab charge variants. Figure 2e confirms the identity of peaks that were made using the deconvoluted intact mass spectrum. It was found that G0F glycoforms are present in 5% relative abundance, followed by G1F and G2F forms constituting about 28% and 16%, respectively. Further, Fig. 2f shows that all the investigated charge variants in this study had the same glycan composition, and thus the observed charge heterogeneity in bevacizumab can be attributed to modifications other than glycosylation. Further, subunit level characterization of the light chain and heavy chain showed that glycosylation is harbored only in the heavy chain while the light chain is devoid of any glycosylation sites (Fig. 2g,h).

In addition, the main bevacizumab product had both of the lysine residues at the C terminus of the truncated heavy chain leading to a − 256 Da shift from the theoretical mass of intact bevacizumab. Relative quantification of the C terminal peptide **SLSLSPG** based on height shows that 95.23% of the species have their terminal lysines clipped. Minor levels of methionine oxidation in residues Met 258 (4.07%), M34 (2.57%), and Met83 (3.62%), all of which are in the heavy chain, are also noted.

### Incomplete clipping of terminal lysine residues in the heavy chain was attributed to the formation of basic 1 and 2 variants

**Figure 3:**
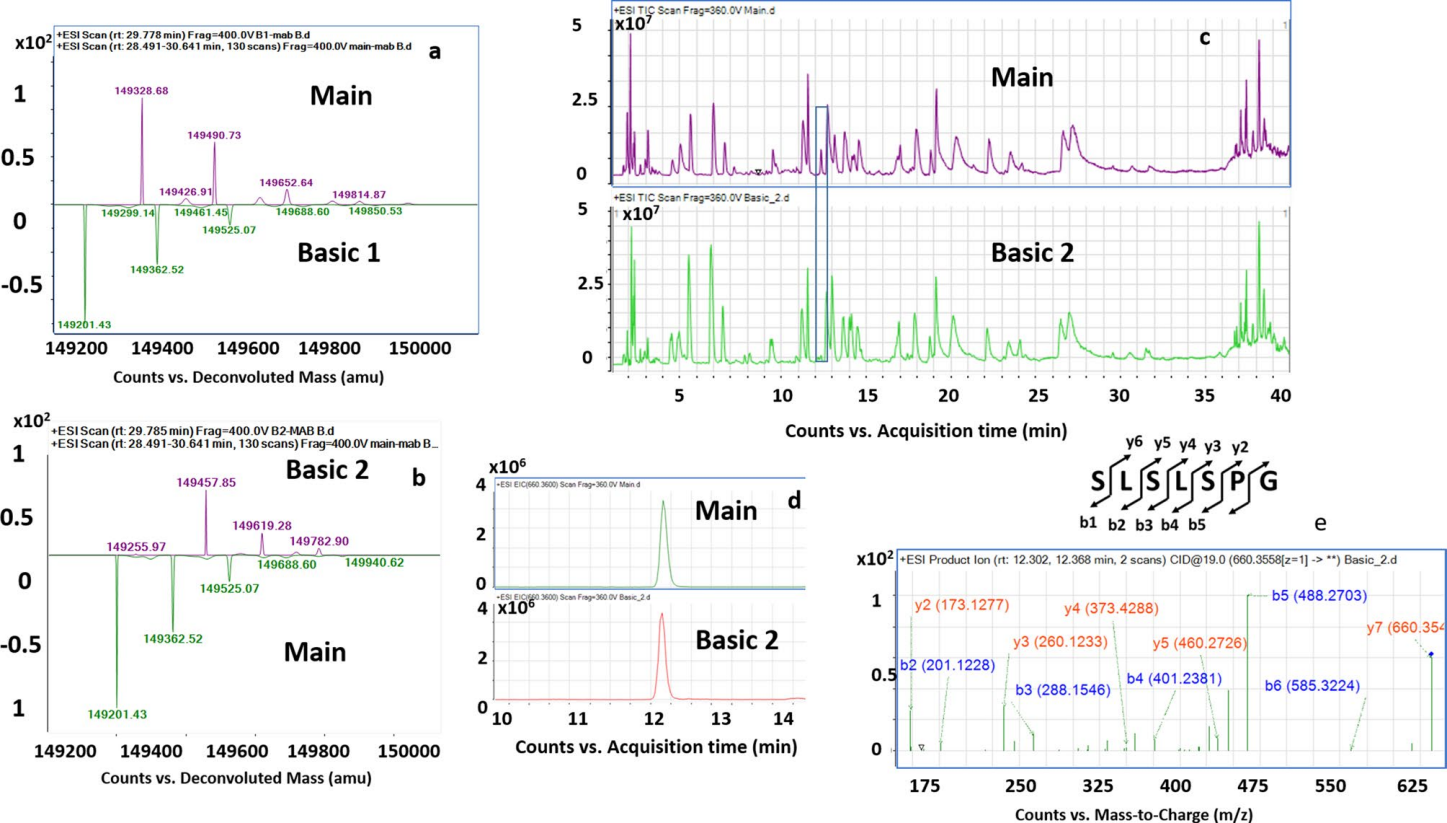
(**a**) Mirror plot of the deconvoluted MS spectrum of basic 1 variant and bevacizumab main product. (**b**) Mirror plot of the deconvoluted MS spectrum of basic 2 variant and bevacizumab main product. Basic 1 variant and basic 2 variant showed an increment of + 128 Da and + 256 Da, respectively, from the expected masses of the G0F, G1F, and G2F glycoforms. (**c**) Stacked view of the total ion chromatogram (TIC) derived from the tryptic peptide mapping of bevacizumab main product and basic 2 variant. The peptide eluting at 12.23 min was found to be significantly low in the basic 2 variant. (**d**) Extracted ion chromatogram (EIC) of peptide eluting at 12.23 min in M+H^+^ ionic form with m/z 660.35 for bevacizumab main product and basic 2 variant. (**e**) MS/MS spectra revealed that the peptide with precursor mass of 660.35 m/z, corresponded to sequence SLSLSPG, and this peptide was formed as a result of C-terminal lysine clipping in the heavy chain of the bevacizumab. The bevacizumab main product had lysine from both the heavy chain clipped, basic 1 variant had lysine from only one of the heavy chains clipped while both lysine were present at the terminal end of the heavy chain in basic 2 variant.

The mass spectrum of the basic peak 1 is perhaps very similar to the main peak, with three different isoforms making up the variant's charge envelope. However, the variant's deconvoluted mass exhibits a consistent mass increase of + 128 Da from the corresponding peaks representing G0F, G1F, and G2F species in the bevacizumab main product (Fig. 3a). The mass shift corresponds to the clipping of C-terminal lysine residues (K453) from one of the heavy chains of the mAb, while the corresponding other heavy chain has the lysine residue preserved at the c terminus end. Relative quantification of the peptide bearing K453 residue based on height shows 53.6% clipping.

Basic peak 2 also exhibits a similar mass spectrum profile as that of the main peak and basic peak 1. However, again, this variant's deconvoluted masses show an increase of + 256 Da corresponding to the masses obtained in the deconvoluted main peak (Fig. 3b). The consistent + 256 Da increase indicates that the both the C-terminal lysine of the heavy chains of the mAb remain intact in the basic peak 2. This observation was confirmed with the tryptic peptide mapping (total ion chromatogram, TIC) that showed the absence of **SLSLSPG** peptide eluting at 12.23 min found in the bevacizumab main product (Fig. 3c). Also, the tryptic peptide map of this peak overlaps with the tryptic digest of the main peak, indicating no other modifications in the variant, except for the two peptides SLSLSPGK (eluting at 9.5 min) and SLSLSPG peptide eluting at 12.23 min (Fig. 3c). The extracted ion chromatogram (EIC) of the peak at 12.23 min (M+H^+^ form with m/z 660.35) shows one order of magnitude difference in the ion abundance between bevacizumab main product and the Basic 2 variant owing to complete clipping of terminal lysine in the former (Fig. 3d). The relative quantification of the peptide SLSLSPG eluting at 12.3 min RT was only 8.23%, while the unmodified peptide was 91.77% based on respective heights. Figure 3e confirms that the Basic 2 variant does not have lysine clipping as the MS/MS spectra of the peptide eluting at 12.23 min and that the peptide sequence is confirmed to be SLSLSPG (i.e., with terminal lysine clipping), which was absent in basic 2 variant.

### Deamidation of N84 residue and untruncated C-terminal lysine resulted in the formation of acidic charge variant

**Figure 4:**
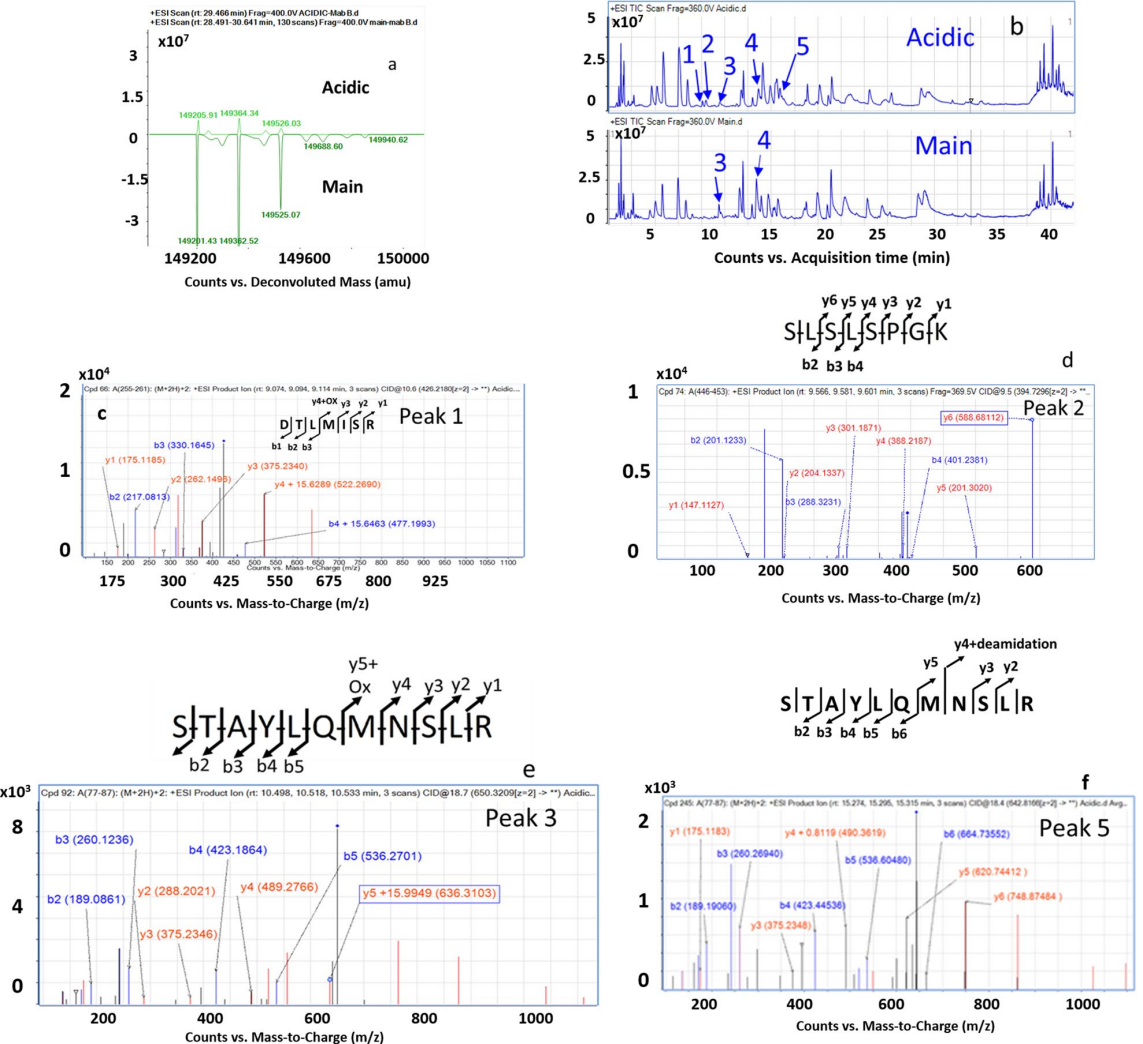
(**a**) Mirror plot of the deconvoluted MS spectrum of acidic variant and bevacizumab main product. An increase of + 4 Da higher mass was observed from the theoretical mass of the G0F glycoform. (**b**) Comparison of the TIC plot of the tryptic peptide map of acidic and unmodified bevacizumab showing differences in respect of five different peaks labeled as peaks 1–5. Peaks 1, 2, and 5 are uniquely present in the acidic variant, while peaks 3 and 4 are present in both the samples, although at different levels. Charge-reduced, isotope-deconvoluted MS/MS spectra of (**c**) DTLIMSR peptide of the acidic variant showing the oxidation of M258 residue (**d**) SLSLSPGK peptide showing intact C-terminal lysine residue (**e**) STAYLQMNSLR peptide showing the oxidation of M83 residue and (**f**) STAYLQMNSLR peptide showing deamidation of the N84 residue in the heavy chain of bevacizumab. The b and y ions of the MS/MS spectra are represented in red and blue color, respectively.

The acidic peak mass spectra exhibited a different profile than that of the main peak. Deconvolution of the mass spectrum yielded predominantly mAb species of masses 149,205.91, 149,364.34, and 149,526.03 Da. The mass shift for these species corresponds to G0F/G0F (+4 Da), G1F/G0F (+1.8 Da), and G1F/G1F (+0.96 Da), respectively (Fig. 4a). However, as the isotopic width of the mAbs are wide (~ 25 Da), it is not reasonable to attribute any modifications resulting from mass shifts less than 25 Da from intact level measurements to a particular variant. Peptide mapping was performed to understand the source of modifications leading to the formation of the acidic variant. Figure 4b shows the TIC of the acidic and main variant. The acidic variant TIC differs from the main variant TIC with respect to five peaks eluting at 9.07 min (peak 1), 9.56 min (peak 2), 10.49 min (peak 3), 12.30 min (peak 4), and 15.27 min (peak 5) retention times. Based on the mass analysis, the four peaks identified correspond to peptide masses: 426.219 for peak 1, 394.729 for peak 2, 660.320 for peak 3, 660.355 for peak 4, and 642.8166 for peak 5. It is noted that while peaks 1, 2, and 5 were uniquely present in the acidic variant, peaks 3 and 4 were present in both the variants at different levels. The peptide masses correspond to the oxidized derivative of the heavy chain peptide 255–261 (DTLMISR) of peak 1, un-truncated C-terminal peptide 446–453 (SLSLSPGK) of peak 2, an oxidized derivative of the heavy chain peptide 77–87 (STAYLQMNSLR) for peaks 3 and the deamidated derivative of the heavy chain peptide 77–89 (STAYLQMNSLR) for peak 5 (Fig. 4c–e). Peak 4 refers to the C-terminal truncated form of the heavy chain peptide 446–453 (SLSLSPG) whose MS/MS spectrum has been shown in Fig. 3e. The relative quantification based on % area indicates that the acidic variant comprises 1.21% of the oxidized derivative of M258 versus 5.67% oxidized levels in the main variant (peak 1). The un-truncated C-terminal peptide SLSLSPGK was absent in the unmodified bevacizumab product and only present in the acidic variant (peak 2). The oxidized derivative of M83 (peak 3) was present in both unmodified and acidic bevacizumab at 12.97% and 3.62%, respectively. The relative levels of peak 4 were estimated to be 95.3% in the main variant and 3.2% in the acidic variant. Peak 5 was noted in the acidic variant but absent in the main variant. MS/MS spectrum of the corresponding peptide indicates deamidation of N84 residue at 85.7% levels in the acidic variant (Fig. 4f). Thus, the observed differences in the levels of the oxidized M258, deamidated N84, and the uncleaved C-terminal lysine in the heavy chain seems to contribute to the observed differences in the charge heterogeneity for the acidic variant.

### Biophysical characterization showed the altered tertiary structure of the acidic variant

**Figure 5:**
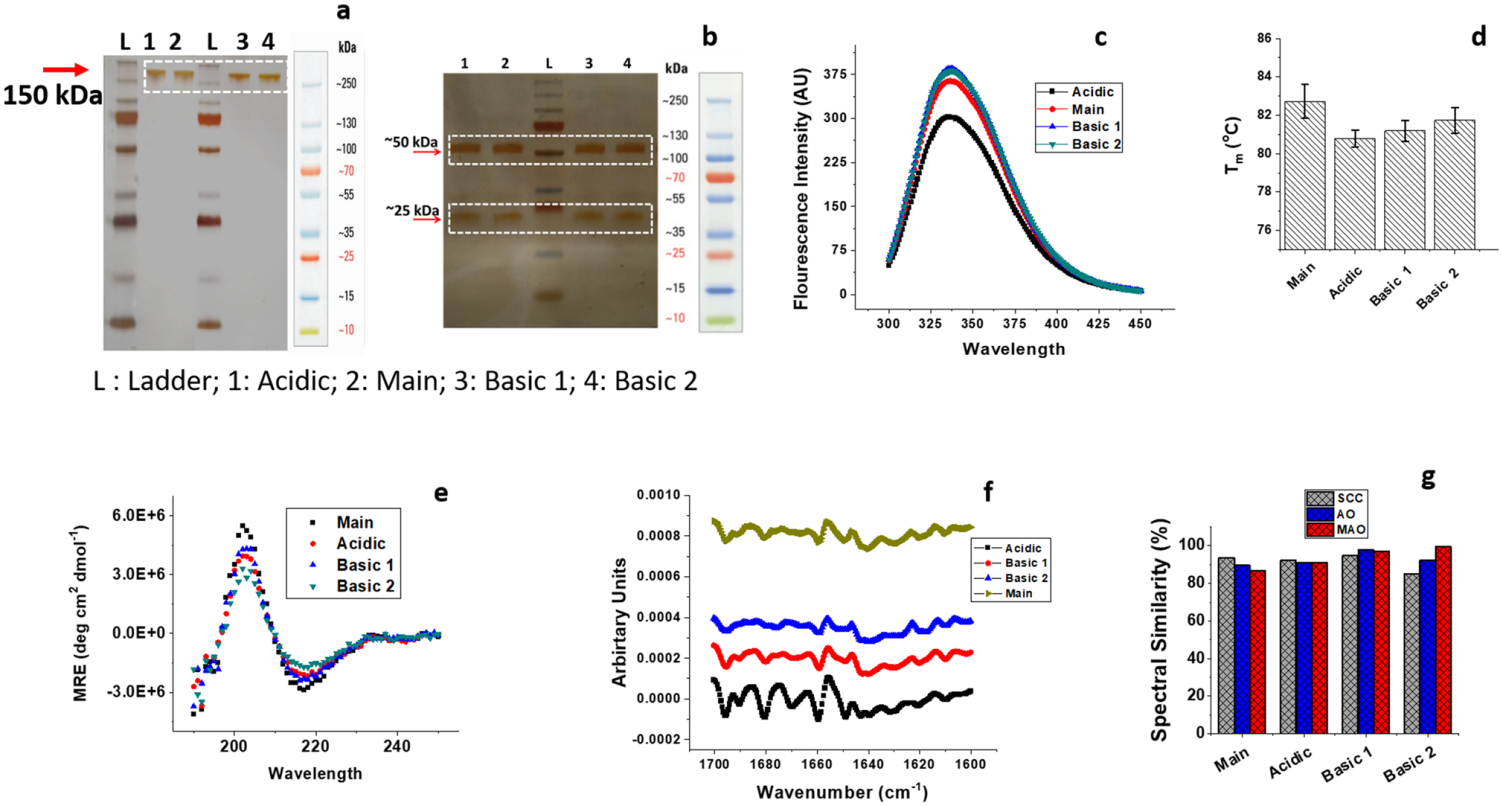
(**a**,**b**) Non-reducing and Reducing SDS PAGE of the bevacizumab main product and the isolated charge variants, white dashed lines identify the cropped region in the respective gel images. Complete scanned gel images for reducing and non-reducing SDS-PAGE are shown in the Supplementary Fig. S2a,b. (**c**) Overlay of the far-UV CD spectra of bevacizumab and the charge variants at a 0.2 mg ml^−1^ concentration. A mean minimum of 218 nm was observed for all the variants suggesting a β-rich composition of the secondary structure. (**d**) Intrinsic fluorescence measurements of the bevacizumab main product and the isolated charge variants with excitation wavelength at 295 nm and emission wavelength recorded between 300 and 400 nm. (**e**) Assessment and comparison of the thermal stability of the charge variants of bevacizumab using fluorescence spectroscopy at a concentration of 1 mg ml^−1^ and an emission wavelength of 337 nm from 55 to 85 °C. (**f**) Second derivative amide I FTIR spectra of the bevacizumab and its charge variants obtained at a concentration of 4 mg ml^−1^. (**g**) Comparison of the spectral features of the samples using statistical algorithms: area overlap (AO), spectral correlation coefficient (SCC) and modified area overlap (MAO).

Once the bevacizumab charge variants' identity was established, the next task was to undertake biophysical and functional attribute assessment of these variants. SDS-PAGE analysis (under reducing and non-reducing conditions) was performed to confirm the purity of the isolated variants and rule out the possibility of proteolytic digestion during sample preparation. Visual observation of the gels revealed that bevacizumab's charge variants display similar behavior vis-à-vis the unmodified bevacizumab. The non-reducing gel does not show the presence of any high or low molecular weight species other than the expected band at ~ 150 kDa for all the samples (Fig. 5a). Under reducing conditions, the samples did not show any heterogeneity, suggesting a similar distribution of disulfide bridges amongst the molecules (Fig. 5b).

### CD spectroscopy

Far-UV CD spectroscopy was used for assessing changes in the secondary structure of the isolated charge variants of bevacizumab. It is seen that the spectra obtained for all charge variants of bevacizumab is similar to a typical spectrum for a monoclonal antibody with a mean minimum at 218 nm and mean maxima at 202 nm (Fig. 5c). The obtained CD spectra of the charge variants suggest a secondary structure rich in β sheet content. It is to be noted that a typical far-UV spectrum of an antibody would exhibit mean maxima at 195nm^30^. The positive deviation (from 195 to 202 nm) in the mean maxima for the bevacizumab samples can be attributed to the increased overall UV-absorbance in the range 190–195 nm with a corresponding increase in the high-tension voltage, which makes the said range less reliable for structural characterization of proteins (Supplementary Fig. S1). All the investigated bevacizumab charge variants exhibited absorbance minima between 217 and 218 nm, characteristic of β sheet rich secondary structure. However, it is noted that the bevacizumab main sample had the largest ellipticity intensity vis-à-vis the charge variants. The Basic 1 variant and acidic variant exhibited a 23% decrease in the ellipticity intensity when compared with the bevacizumab main sample, while basic 2 variant displayed a 50% decrease in the ellipticity intensity. The observed changes in the ellipticity intensity were significant as the values exceeded the typical range of 5–10% error associated with measuring the secondary structure of proteins^31^.

### Fluorescence spectroscopy

The 3D structural integrity of the charge variants was compared with the main bevacizumab product using fluorescence spectroscopy. The emission maxima of all the samples were observed at 335 ± 2 nm, indicating a slightly open structure (Fig. 5d). The obtained emission maxima is consistent with the reported values in the literature for bevacizumab^19^. However, similar to the CD profiles, a difference in the fluorescence emission intensities across the charge variants were noted. It is known that the quantum yield of a fluorophore in a hydrophobic environment (tightly packed state) is generally higher as compared to that in the hydrophilic environment (solvent-exposed state)^19^.

Figure 5d clearly shows that the samples' fluorescence intensities follow the order: Basic variant 1 = Basic variant 2 > Main > Acidic variant. The higher fluorescence intensities of Basic variants 1 and 2 correspond to a more tightly packed structure and, therefore, clearly stand out compared to the other basic bevacizumab variants in terms of structural integrity. On the contrary, the acidic variant's relatively low fluorescence intensity indicates some conformational change that has induced flexibility in certain regions of the protein that otherwise was buried in the core and hence were inaccessible. Thus, the acidic variant exhibited a similar secondary structure vis-à-vis the main bevacizumab but differed considerably in terms of the tertiary structure.

Further, the bevacizumab charge variants' stability was examined by thermal denaturation of the variants and monitored using fluorescence spectroscopy. The fluorescence intensity versus temperature curves of each charge variant was fitted to a Boltzmann function to derive the respective melting temperature. It can be clearly seen that the denaturation process of all the charge variants and main bevacizumab follows a two-state model indicated by a minimum and a maximum in the thermogram's fluorescence intensity (Supplementary Fig. S3a–f). The charge variants' melting temperatures were calculated with acceptable reproducibility of < 0.5 °C (Fig. 5e). It was observed that the charge variants' melting temperatures are in the range of 79.3–82.7 °C and are not significantly different. The single transition in the fluorescence signal indicates that the unfolding of bevacizumab and its charge variants proceeds globally, unlike other monoclonal antibodies in which the subunits unfold independently and gives rise to multiple transitions^28^. The increasing fluorescence signal beyond ~ 80 °C is a result of irreversible aggregation evidenced from residual precipitates that remained in the cuvette after cooling to room temperature. Thus, the conformational changes in the charge variants do not appear to affect the molecule's stability due to underlying modifications.

### Time-resolved fluorescence spectroscopy

The fluorescence lifetime distribution and associated pre-exponential factors were determined by fitting the time-resolved intensity decay of bevacizumab and its charge variants to two discrete exponential functions (Supplementary Table S1; Supplementary Fig. S4). As such, two fluorescence lifetimes in the picosecond and nanosecond scale, respectively, were obtained for each sample. The fluorescence lifetimes of the Basic variants 1 and 2 and the acidic variant were comparable to that of the bevacizumab main product. The fraction of molecules exhibiting nanosecond scale fluorescence was 82.4%, 86.1%, 84.5%, and 81.6%, respectively, while the fraction of molecules exhibiting picosecond scale fluorescence lifetimes were 17.5, 13.6, 15.4, and 18.3%, respectively. Thus, in terms of the local environment around the aromatic residues in these samples, it can be concluded that meaningful differences were not observed.

### FTIR spectroscopy

Figure 5f shows the second derivative FTIR spectrum of bevacizumab and its charge variants. The amide I frequency peaks corresponding to antiparallel β-sheet structure (1635 cm^−1^ and 1697 cm^−1^), intermolecular β-sheet (1617 cm^−1^), β-turns (1668 cm^−1^ and 1682 cm^−1^), and random coils (1650 cm^−1^) were chosen as per the literature, and a comparison was made between the charge variants and the unmodified bevacizumab product^30^. The main product's spectra, Basic variant 1, Basic variant 2, and acidic variants, were dominated by the band at the 1635 cm^−1^, suggesting intramolecular native β-sheets as the major structural component. It can be surmised that the acidic variant, unmodified product, and Basic variants 1 and 2 have similar secondary structural organization.

Further, a quantitative comparison of the spectral similarity of the amide I FTIR spectra between the charge variants with the unmodified product was performed using advanced statistical algorithms such as spectral correlation coefficient, area overlap, and modified area overlap, respectively (Fig. 5g). All the charge variants of bevacizumab exhibited remarkable similarity with the unmodified product. Thus, based on the data obtained, it can be inferred that basic variants 1 and 2, acidic variant, and bevacizumab main product offer similar secondary structures.

### Cell proliferation assay showed equivalent activity for lysine variants and reduced activity of the acidic variant

**Figure 6:**
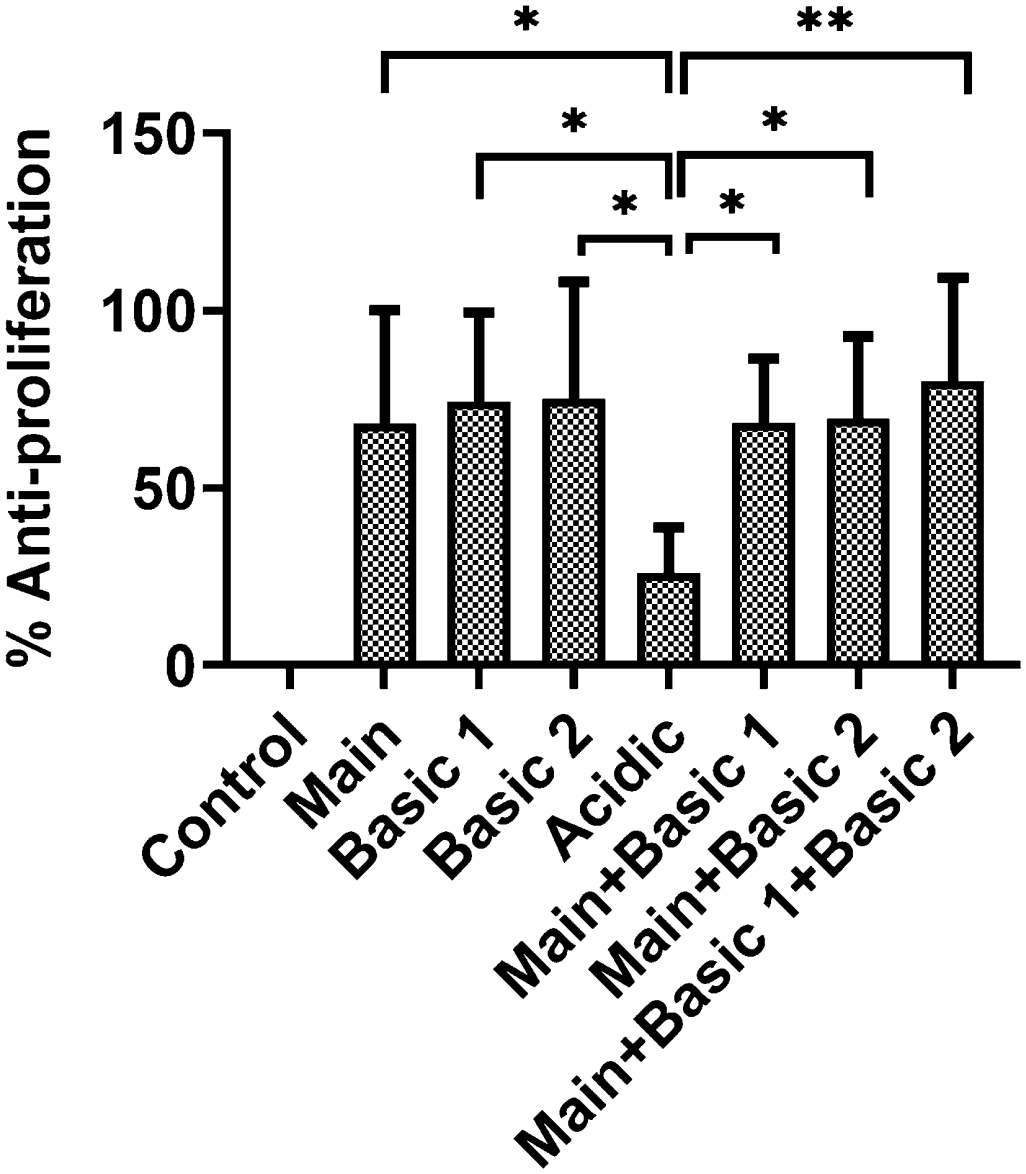
The anti-proliferative activity of bevacizumab main product and its charge variants on HUVEC cells. Rituximab was used as a control as it does not show binding with VEGF. The cell readout was taken using absorbance at 490 nm using the MTS-PMS assay. (**indicates p < 0.005, ** indicates p < 0.01).

The mechanism of action of bevacizumab is via inhibition of VEGF-A induced proliferation of endothelial cells. The isolated charge variants of bevacizumab were tested on HUVEC cells for their anti-proliferation activity using the MTS-PMS assay^32^. It was found that basic variants 1 and 2 exhibited similar anti-proliferation activity than the unmodified product (Fig. 6). However, the acidic variant had lower activity in inhibiting HUVEC cells' proliferation compared to the unmodified product. Also, the acidic variant's anti-proliferative activity was significantly less than both basic variant 1 and basic variant 2. It was noted that basic variant 1 and basic variant 2 have comparable activity. Based on this observation, we pooled these two variants and the main product in the ratio 1:1. We found no significant changes in the anti-proliferative activity versus the unmodified product implying equivalent potency of basic variant 1 and basic variant 2 with the main product. Moreover, as expected, the acidic variant showed significantly lesser anti-proliferative activity than the pooled basic variants along with the main product.

## Discussion

Multiple enzymatic and non-enzymatic modifications such as deamidation, oxidation, glycation, and C-terminal lysine clipping, contribute to charge heterogeneity observed in monoclonal antibody products^15^. The differences in the levels of these variants during commercial manufacturing serve as an indicator of process consistency, and therefore, any observed differences in the relative proportions may impact stability and biological activity of the target molecules^13^. These differences may pose a severe challenge for biosimilar development, especially in demonstrating product comparability.

One of the impediments in performing a comprehensive evaluation of the impact of charge variants on safety and efficacy is our limited ability to isolate individual charge variants in quantities required for their extensive biophysical and functional characterization^6^. Capillary zone electrophoresis (CZE), imaged capillary isoelectric focusing (cIEF), and ion-exchange chromatography can achieve satisfactory separation of the variants, but fail to generate sufficient material for detailed characterization^14^. Weak exchange chromatography (WCX) has been proposed as a viable alternative by allowing simultaneous separation, analysis, and sample preparation^33^. However, WCX suffers from the disadvantage of high sensitivity to the pH based separation methods due to the varying pKa of the carboxylic acids (common ligands in WCX resins) as a function of pH^34^. On the contrary, since strong cation exchange chromatography (SCX) involves the use of sulphonic acid groups as ligands, the sensitivity to pH gradient is significantly reduced, and consistently binding capacities over a range of operating conditions can be achieved^35^. In the present work, we have employed semi-preparative strong cation exchange chromatography to isolate charge variants (in purity > 85%) for structure, stability, and functional characterization of mAb charge variants.

For mAb products, the first two basic variant peaks constitute > 80% of the overall basic variant content^14,36–38^. Typically, incomplete removal of the C-terminal lysine residues is the cause of the formation of these variants^36^. The present study with bevacizumab also establishes that these variants are formed by incomplete removal of either one (basic 1 variant) or both lysine residues (basic variant 2) C terminus of the bevacizumab heavy chain. A previous characterization study has demonstrated similar conformation, solution dynamics, chemical denaturant-induced unfolding profile, and lysine variants' stability vis-à-vis the unmodified product^39^. Also, it is shown that terminal modifications in mAbs often do not substantially impact the structure, stability, or function as these regions are highly exposed and do not directly participate in the target binding directly^40^. The present study's findings also agree with these reports about the structural conformation of the bevacizumab lysine variants.

It has been reported that the lysine variants of mAb exhibit enhanced binding affinity to FcRγIIIa and FcRn receptors^14^. While the former is implicated in exerting the biological activity, the latter mediates the prolonged serum half-life of IgGs^41^. The enhanced half-life could translate into improved in vivo efficacy. Therefore, we tested this hypothesis by assessing the biological activity of the lysine variants alone and combining the unmodified product in the ratio 1:1. It is seen that while the lysine variants exhibited similar structural as well as biological activity as that of the main unmodified product, there was no evidence of improvement in the activity upon enriching the final product pool with the lysine variants of bevacizumab.

**Figure 7:**
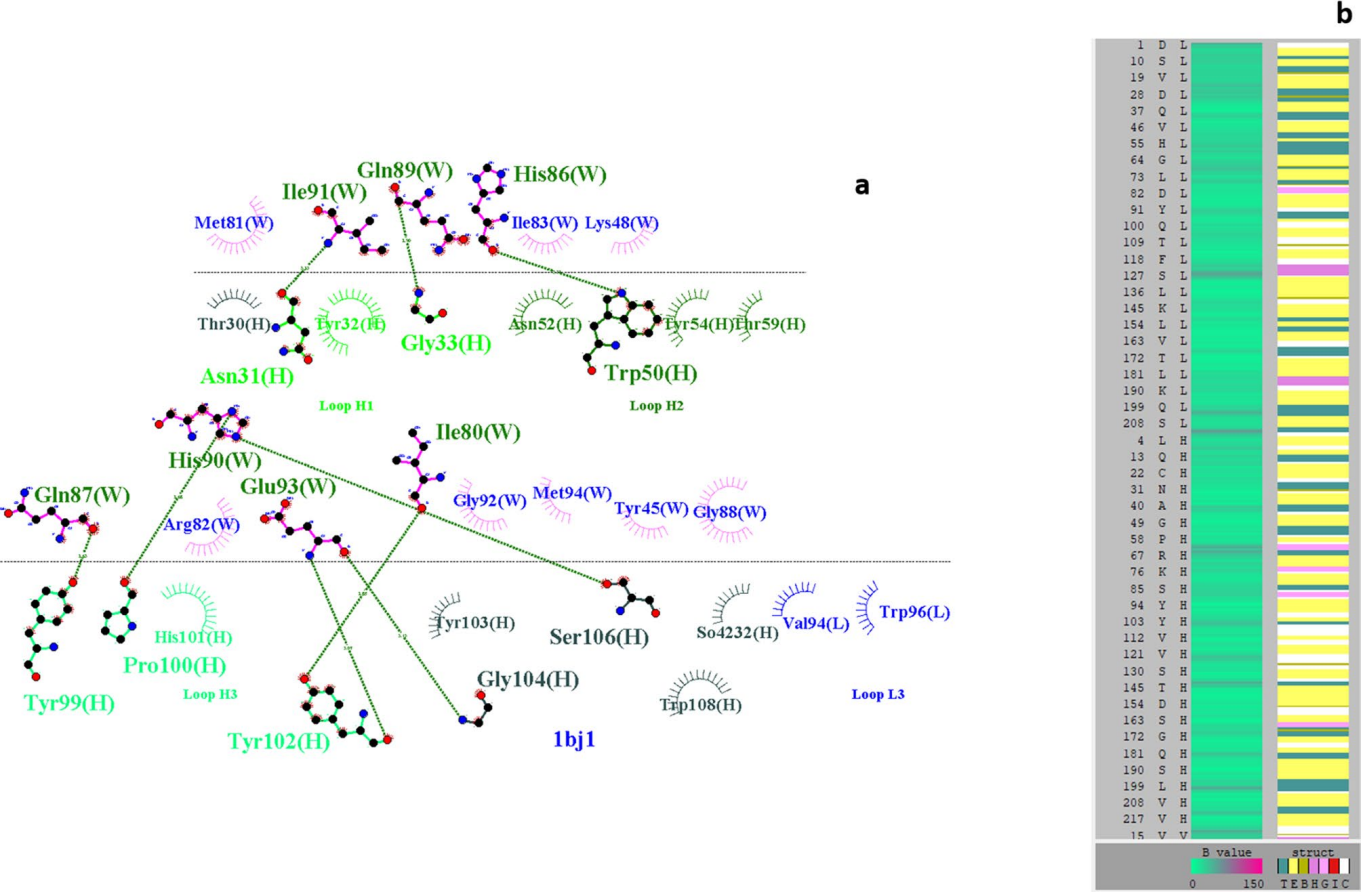
(**a**) LIGPLOT of the interface between the bevacizumab: VEGFR in PDB 1BJ1. The N84 residue (deamidated) in the acidic variant is not involved in the interaction with VEGFR. Similarly, M258 and M83 (Oxidized) are also not involved in binding interaction with VEGFR. (**b**) Listing amino acids in the light chain and heavy chain in terms of their secondary structure distribution. Notations used are as follows: *T* turn, *E* extended conformation, *B* isolated bridge, *H* alpha helix, *G* 3/10 helix, *I* Pi-helix, *c* coiled. The deamidated N84 residue is solvent-exposed and is located in a turn secondary structural conformation.

However, unlike the first two basic variants with known modifications (incomplete lysine removal), the acidic variants in mAbs could be formed as a result of higher levels of sialic acid content^42–44^ or deamidation of Asn residues to IsoAsp or Asp residues that are located either in the Fc region or the complementary determining region (CDR) of the mAb^45–47^. CEX separation of mAbs with IsoAsp or Asp residues on the same position indicates that they elute at different retention times, suggesting a conformational difference between the two that affects charge distribution on the protein and thereby its interaction with the CEX resin^15^. In the case of the commercial bevacizumab biosimilar analyzed in this study, the acidic variant constituted about 6% of the total charge variant composition of the product. Deamidation of N84 residue in the heavy chain and preserved c-terminal lysine residue in the heavy chain (unlike that of the main variant having both C-terminal lysine residues clipped) were attributed to the formation of the acidic variant in bevacizumab. Additionally, subtle differences noted regarding the tertiary structure and stability resulted in their lower antiproliferative activity on HUVEC cells. The bevacizumab's primary sequence indicates that a serine residue follows the acidic variant's deamidation site (N84). It is known that the deamidation of asparagine residue at neutral or alkaline pH is facilitated if the amino acid next to either does not have a side chain (e.g., Glycine) or, if present, is small such as Serine. Further, an analysis of the crystal structure of bevacizumab in complex with VEGF (PDB: 1BJ1) shows that N84 is highly solvent-exposed in a turn region and does not share any interaction with the neighboring side chains, fulfilling all conditions for the residue in becoming a potential deamidation hotspot (Fig. 7a,b).

Apart from lysine and deamidation variants, other modifications that contribute to the mAb charge heterogeneity include methionine oxidation^49^, isomerization of Asp to IsoAsp^50^, incomplete disulfide bonds^51^, aglycosylation^52^, fragmentation^53^, and aggregation^42^. These modifications are generally located within the molecule and are found to have structural and functional implications. For instance, a Fab molecule with unpaired disulfide bonds was found to exhibit only ~ 28% potency vis-à-vis the Fab containing the fully formed disulfide bonds^54^. Similarly, mAb with succinimide in the heavy chain CDR had only 30% of the biological activity compared to the corresponding mAb with original Asn at the same position^55^. In contrast, succinimide located in the light chain CDR of another mAb did not show any difference in biological activity compared with the wild type mAb with usual Asp residue.

Methionine oxidation in the CH2 domain of the mAb causes conformational changes that decrease thermal stability and increase aggregation propensity^56^. In the present study, basic variants 3 and 4 exhibited an altered structure. However, basic 3 variant displayed reduced activity while the basic 4 exhibited comparable activity as the unmodified bevacizumab (Supplementary Fig. S5a–c). The near-comparable activity to the unmodified bevacizumab of the basic variant 4 is likely because the modification is perhaps in the Fc region that does not hamper the activity. The underlying modifications contributing to the formation of basic variant 3 and basic variant 4 were fragmentation and methionine oxidation (Supplementary Figs. S6, S7). There are many reports on the differential impact of methionine oxidation on the structure and function of proteins, and as such, it is not surprising to observe the fully functional form of basic variant 4^57–59^. Owing to fragmentation, basic variant 3 lost the structural integrity needed for full biological activity, but this requires further investigation.

Analytical comparability between the innovator and biosimilar is a critical part of biosimilar development^60^. In this regard, closely matching heterogeneities between products is a crucial task. As demonstrated in this paper, it is evident that matching charge heterogeneity is complicated because the same modifications (for instance, deamidation) at two different locations result in an altogether different impact on function. Hence, the variants' analytical isolation and performing their safety and efficacy evaluation are necessary for the development of a safe and efficacious biosimilar product.

## Conclusions

The current industry paradigm in biosimilars development is to closely match its quality attributes to the innovator to the extent possible. Often the specification for the charge variant composition for an innovator and biosimilar products is based on the cumulative amount of acidic and basic variants and not for each charge variant. As seen in the study presented here, different variants are likely to have different structural conformations and biological activity. Hence, innovators need to understand these implications before deciding which charge variants should be pooled into the final product. Similarly, for biosimilar producers, this knowledge is essential to establish adequate process controls so that the final product matches the innovator product's charge heterogeneity.

## Materials and methods

### Materials

The bevacizumab harvest obtained in CHO cell line was donated to us by a major Indian biopharmaceutical manufacturer. All other chemicals used in the study were procured from Sigma Aldrich unless otherwise stated.

### Isolation of bevacizumab charge variants

Protein A elute of mAb B was diluted to 2 mg ml^−1^ and loaded on YMC 10 biopro column (10 µm particle size) on the AKTA Avant chromatography system from GE Healthcare, Uppsala, Sweden. The buffer used 15 mM phosphate, 35 mM NaCl, pH 6 (A) and 15 mM phosphate, 15 mM Tris, 35 Mm NaCl, pH 9 (B). A gradient of 0–100% B was applied for 15 column volumes at a flow rate of 0.2 ml min^−1^ for elution. The output profile was monitored online using UV absorbance at 280 nm. Fractions of 1 mL each were collected.

### Purity analysis of the isolated fractions

The collected fractions were analyzed by cation exchange chromatography for the purity of individual charge variant species using a previously established and validated method^38^. Briefly, the mobile phase comprised of the 15 mM phosphate buffer (A, pH 6.2, conductivity 2.2 mS cm^−1^) and 150 mM phosphate buffer (B, pH 6.2, conductivity 12.4 mS) with 0.05% sodium azide. A sigmoidal gradient described in the paper^38^ was applied on MAbPac SCX-10, RS, 5 m, 250 × 4.6 mm column with a flow rate of 1 ml min^−1^. UV absorbance was recorded at 215 nm, with the column temperature maintained at 28 °C throughout the run. Size exclusion chromatography (SEC) was used to analyze the aggregate content in each isolated fraction. SEC analysis was performed using 50 mM phosphate with 300 mM NaCl (pH 6.8) as mobile phase on Mabpac SEC-1, 5 µm, 300 × 4 mm column at a flow rate of 1.5 ml min^−1^. UV absorbance at 215 nm was recorded. The analyzed fractions were pooled so that a particular mAb variant (main, acidic, or basic) was present in amounts with purity > 95%.

### Intact MS analysis of the charge variants

The pooled fraction at a 0.4 mg ml^−1^ concentration was buffer exchanged into 0.1% formic acid using 10 kDa MWCO centricon (Pall Corporation, USA). Intact mass measurements on these fractions were made on AdvanceBio RP mAb C4 (4.6 × 100 mm, 5 μm, Agilent Technologies) column operated at 80 °C using Agilent 1290 Infinity Quaternary LC system coupled online to Agilent 6230 ESI-TOF–MS. 5 μg sample was loaded on the column and separated using a 35 min linear gradient from 2 to 60% B at a flow rate of 0.5 ml min^−1^. Detection was performed by monitoring UV absorption at 280 nm and TIC was recorded for 1000–6000 m/z. MS spectra were calibrated in the positive ion mode before analysis. The capillary gas temperature/voltage (Vcap) was set to 350 °C and 5500 V, respectively, and the fragmentor voltage (Vfrag) was 400 V. The MS spectra were deconvoluted using the maximum entropy (MaxEnt) algorithm with resolution of 20,000 and 8 iterations.

### Subunit MS analysis of the charge variants

The subunit analysis of the bevacizumab was carried out by separating the light chain and heavy chain. The sample preparation involved solubilizing the isolated charge variants (50 µg) with 30 µl of 6 M guanidine followed by the addition of 3 µl of 0.5 M DTT to a final concentration of 50 mM. The samples were reduced by incubation at 55 °C for 1 h and finally equilibrated to room temperature before subjecting to LC–MS analysis^43^. LC–MS analysis consisted of separating the light chain and the heavy chain of bevacizumab charge variants on an Agilent AdvanceBio RP mAb C4 (4.6 × 100 mm, 5 μm, Agilent Technologies) column operated at 60 °C using Agilent 1290 Infinity Quaternary LC system coupled online to Agilent 6230 ESI-TOF–MS. The mobile phase used for the separation consisted of buffer A (water + 0.1% formic acid) and buffer B (Acetonitrile + 0.1% formic acid) at a linear gradient from 2%B to 65%B in 30 min. MS spectrum was acquired in the mass range 500–3000 Da in positive mode. Other MS parameters included capillary gas temperature/voltage (Vcap) was set to 250 °C and 4500 V, respectively, and the fragmentor voltage (Vfrag) was 280 V. The MS spectra were deconvoluted using the maximum entropy (MaxEnt) algorithm with resolution 20,000 and 9 iterations.

### Peptide mapping analysis

The mAb charge variants after tryptic digestion were analyzed using peptide mapping on an Agilent AdvanceBio peptide mapping column C18 (4.6 × 150) mm employing the following gradient of mobile phase A (0.1%formic acid in water) and mobile phase B (0.1% formic acid in acetonitrile): equilibration with 5%B for 2 min, 2–35% B till 35 min, 35–90%B till 37 min and then holding at 90% B till 40 min at 0.4 ml min^−1^. A post-time equilibration at initial %B was performed for 5 min. MS was performed in positive ion mode in the mass range m/z 100–1700, with an MS scan rate of 4 spectra s^−1^ and MS/MS scan rate of 3 spectra s^−1^. MS/MS isolation width of ~ 4 amu was used.

### Glycan analysis

Labeled N-glycan samples were prepared using the AdvanceBio N-glycan sample preparation kit using vendors' recommended protocol. HILI analyses were performed on a 1260 Agilent LC system equipped with an Agilent 1260 Infinity Fluorescence Detector (G1321B). The detector was set to λ_Ex_ = 285 nm, λ_Em_ = 345 nm, with PMT gain = 10. Glycans were chromatographically separated with an AdvanceBio Glycan Mapping column (2.1 × 100 mm, 1.8 µm) according to the method recommended by the vendor.

### SDS-PAGE

Bevacizumab and its charge variants were run on a Sodium Dodecyl sulfate–polyacrylamide gel electrophoresis (SDS-PAGE) gel under reducing and non-reducing conditions. A 12% resolving gel was used under reducing conditions, and 8% gel was used under non-reducing conditions. . Samples were denatured in boiling water for 15 min with loading buffer in the presence (reducing condition) and absence (non-reducing condition) of a reducing agent. 1 µg of the sample was loaded onto the gel, and the experiment was carried out in a tris–glycine running buffer with a PageRuler Plus Prestained Protein Ladder, 10–250 kDa (Thermo Fisher). The experimental parameters used were: stacking voltage of 80 V for 30 min and separating voltage of 120 V for 120 min (under both reducing and non-reducing conditions). Staining was done by silver staining protocol. The gel was briefly fixed with methanol:acetic acid solution (50%:12%), following which it was impregnated using sodium thiosulphate solution and stained using 0.2% silver nitrate solution.

### CD spectroscopy

Far-UV CD spectra of the isolated charge variants were taken in the range of 190–250 nm at 25 °C on a JASCO J-815 spectropolarimeter with a spectral bandwidth of 5 nm and using a 0.1 cm path length quartz cell (for far-UV) at a scan speed of 50 nm min^−1^. Three spectra were recorded of the sample (0.2 mg ml^−1^, for far-UV), were averaged, and finally plotted after subtracting the buffer baseline. Mean residue ellipticities (MRE, deg cm^2^ dmol^−1^) were calculated, where M_o_ is mean residue weight (112.12 Da residue^−1^), θ is observed ellipticity (mdeg), l is the light path (cm), and c is the concentration (mg ml^−1^)^19^.

1 $${\text{MRE}} = \frac{Mo \times \theta }{{10 \times l \times c}}$$

### Fluorescence spectroscopy

Fluorescence spectra were determined on a Perkin-Elmer LS-5 fluorescence spectrophotometer at 25 °C using a 0.5 cm cuvette. The slit widths were adjusted at 10 nm for excitation and 5 nm for emission. The isolated charge variants were diluted from the stock solution to a final protein concentration of 0.2 mg ml^−1^ and incubated at room temperature for l0–15 min prior to recording the spectra. The excitation wavelength was 295 nm, while the emission spectra were recorded from 300 to 400 nm. The spectrum for each sample was recorded in triplicates, and the average values obtained were plotted against time.

The thermal stability of the charge variants of bevacizumab was determined at a concentration of 1 mg ml^−1^ by recording fluorescence intensity of emission fixed at 337 nm from 55 to 85 °C. The rationale for choosing this temperature range was that no transitions in the emission spectra were observed prior to 55 °C. The spectra were recorded in a 1 mm path length cuvette, and the temperature was increased at a rate of 1 °C min^−1^. The buffer's signal was subtracted, and the sample spectrum smoothed using 11 point Savitsky–Golay and normalized using MATLAB. The melting temperature was obtained by fitting the Boltzmann function to the thermal denaturation data.

### Time resolved fluorescence spectroscopy

The fluorescence lifetimes of the mAb charge variants at a concentration of 0.8 mg ml^−1^ were measured on a time-correlated single-photon counting (TCSPC) setup (FL920, Edinburgh Instruments, UK). The bevacizumab charge variants were excited with a wavelength of 280 nm using a light emitting diode, 200 ns FWHM with PPD and laser diode, and the respective decay profiles mAb charge variant were monitored at 337 nm at the magic angle (54.7 °C). A solution of colloidal silica (Ludox) was inserted into the buffer to acquire the instrument response function (IRF). The IRF was used to deconvolute the fluorescence time-resolved decay of the isolated charge variants. The decay times (Ʈ) were obtained by using Marquardt least-squares minimization and fitting a four-parameter fit to the decay profile of the charge variants. The decay function was assumed to be the sum of two exponentials given by the equation:

2 $$F\left( t \right) = \mathop \sum \limits_{i} \propto_{i} \exp (t/\tau_{i} )$$

where F(t) is the experimental intensity decay, ${\propto }_{{\varvec{i}}}$ is the pre-exponential factor, and ${{\varvec{\tau}}}_{{\varvec{i}}}$ is fluorescence lifetime of the ith discrete component, both recovered upon fitting.

The fractional intensity (f_i_) of each lifetime and the mean lifetimes <${{\varvec{\tau}}}_{{\varvec{i}}}$> are given by Eq. (3) and (4), as follows:

3 $$f_{i} = \frac{{ \propto_{i} \tau_{i} }}{{\mathop \sum \nolimits_{i} \propto_{i} \tau_{i} }}$$

4 $$\left\langle {\tau_{i} } \right\rangle = \mathop \sum \limits_{i} f_{i} \tau_{i}$$

### FTIR spectroscopy

Attenuated total reflectance-Fourier transform infrared spectroscopy (ATR-FTIR) spectra of the bevacizumab charge variants were acquired on a Thermo Scientific Nicolet iS50 FTIR Spectrometer equipped with an attenuated total reflectance (ATR) accessory that makes use of a monolithic diamond crystal. The spectra were collected with 256 scans and a 4 cm^−1^ resolution in the region of 600–4000 cm^−1^ and a set of three replicates of each charge variant of bevacizumab was analyzed at a concentration of 4 mg ml^−1^. A second derivative with 13 points Savitsky-Golay smoothening was made. A baseline adjustment using 3–4 point correction was made in the Amide I region (1600–1700 cm^−1^). Finally, the spectra of all the charge variants of bevacizumab was area normalized for comparison. The area normalized spectra of the bevacizumab charge variants were used for spectral similarity analysis using area overlap (AO), spectral correlation coefficient (SCC) and modified area overlap (MAO) algorithms between the main bevacizumab product and the isolated charge variants. All the results are shown in terms of percentage and is correlated with spectral similarity.

The spectral correlation coefficient (SCC) is calculated using the following equation:

5 $$SCC = \frac{{\mathop \sum \nolimits_{N} x_{i} y_{i} }}{{\sqrt {\sum x_{i}^{2} \sum y_{i}^{2} } }}$$

where x_i_ and y_i_ represent the spectral absorbance values of the unmodified product and the charge variant spectra at the ith frequency position. SCC value lies between 0 and 1 where 0 indicates no similarity between spectra, and 1 indicates spectra that are identical.

The area overlap between the unmodified bevacizumab product and the charge variants were calculated using the following equation:

6 $$\% Area \, Overalp = \left( { \frac{{\sum Area_{overlap} }}{{\sum Area_{reference} }}} \right) \times 100$$

Modified area overlap was calculated using the equation:

7 $$S_{MAO} = \left( {S_{2ndderiv. } } \right)^{2}$$

where S_2ndderiv_ is the signal of the 2nd derivative spectrum at a given wavenumber.

### Cell proliferation assay

The cell proliferation was tested by performing MTS-PMS assay^32^. Briefly, HUVEC cells were seeded in a 96-well plate at a density of 10,000 cells well^−1^ and incubated for 48 h with 0.1 mg ml^−1^ of bevacizumab charge variants. MTS-PMS in the ratio 10:1 was added, and plate readout was taken at 490 nm.

8 $$\% Cell \;proliferation = \frac{Absorbance \;of\; treated \;cells }{{Absorbance \;of\; untreated \;cells}} \times 100$$

The cell proliferation of the charge variants was reported as the % proliferation with respect to the standard bevacizumab.

### Statistical analysis

The statistical analysis was performed in the GraphPad Prism software ver.6. One way analysis of variance (ANOVA) Tukey test was used to compare the sample groups and the control, wherein p < 0.05 was considered as the parameter signifying significant difference. The results are expressed as the mean ± standard deviation from each experiment performed in triplicate.

## Supplementary Information

Supplementary Information

## Data Availability

All the raw data of this study is available with the corresponding author upon request.
