# Supplementary material for: LC–MS based case-by-case analysis of the impact of acidic and basic charge variants of bevacizumab on stability and biological activity
Source: Sci Rep. 2021 Jan 29;11:2487. doi: 10.1038/s41598-020-79541-2 (PMC7846745; doi:10.1038/s41598-020-79541-2)
Supplement: Supplementary file 1 — Supplementary Information [file 41598_2020_79541_MOESM1_ESM.pdf]

Supplementary Information

LC-MS based case-by-case analysis of the impact of acidic and basic charge variants of bevacizumab on stability and biological activity

Sumit Kumar Singh[1], Deepak Kumar[1], Himanshu Malani[1], Anurag S. Rathore[1*]

Department of Chemical Engineering, Indian Institute of Technology, Hauz Khas, New Delhi, India

[*]Corresponding author:
Prof. Anurag S. Rathore
Department of Chemical Engineering
Indian Institute of Technology
Hauz Khas, New Delhi, 110016, India
Phone: +91-9650770650
Email: asrathore@biotechcmz.com
Website: www.biotechcmz.com

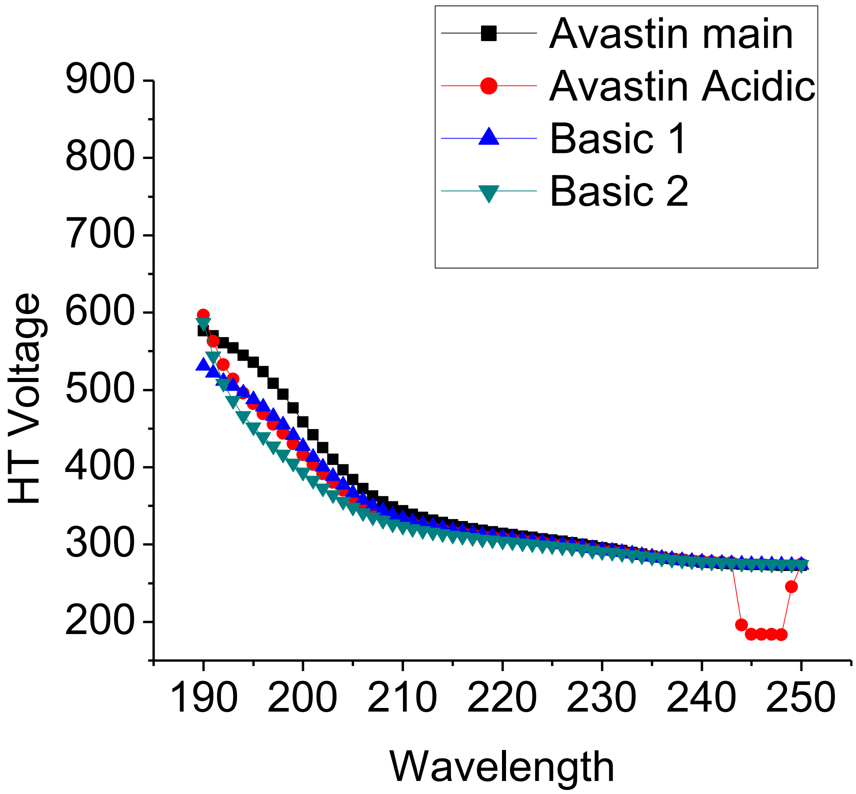

Supplementary Figure 1: High tension CD voltage as function of time showing considerable changes in the HT voltage in the 190-195nm region making the interpretation about the structural architecture of the variants using these wavelength region ambiguous.

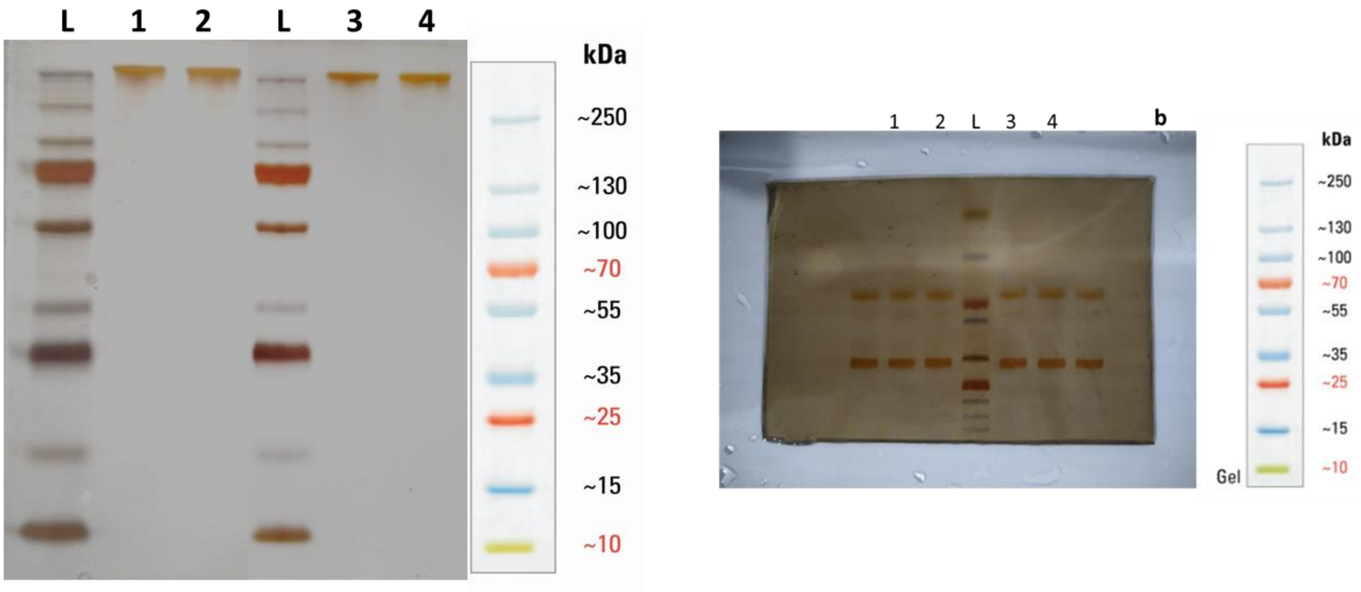

L : Ladder; 1: Acidic; 2: Main; 3: Basic 1; 4: Basic 2

Supplementary Figure 2: a) Complete scanned gel images for non-reducing and reducing SDS-PAGE of the figure 5a, 5b.

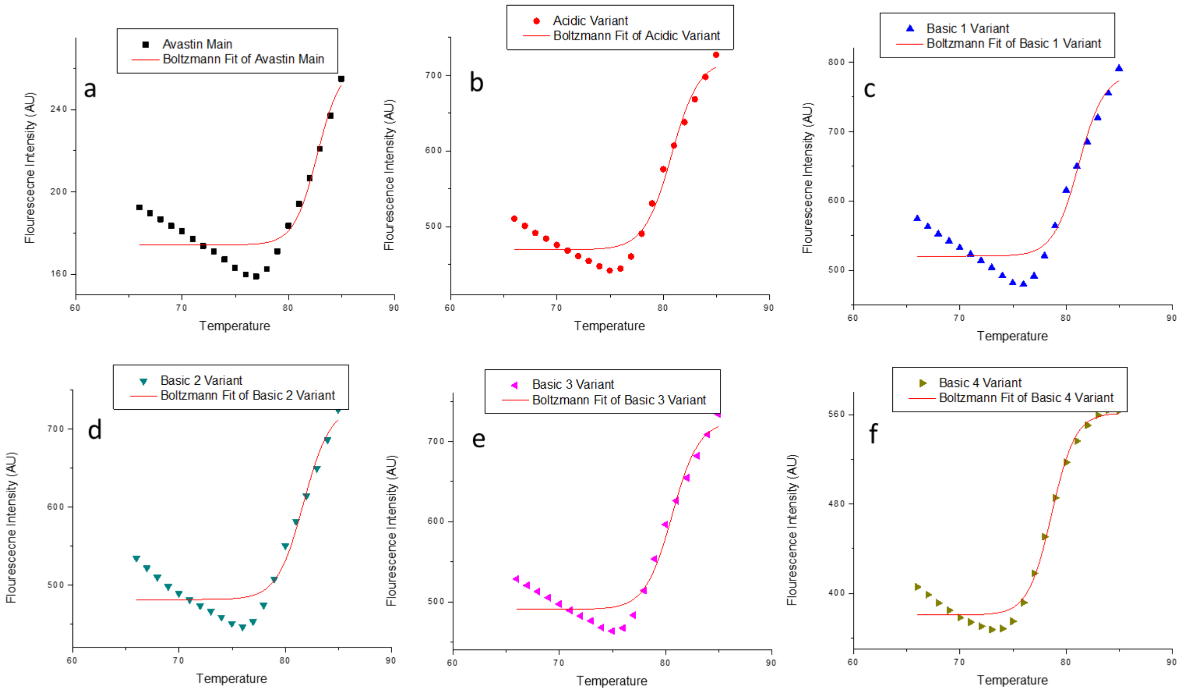

Supplementary Figure 3: Assessment of the thermal stability of the charge variants of bevacizumab using fluorescence spectroscopy at a concentration of 1mg/ml and emission wavelength of 337nm from 55°C to 85°C. The melting temperature were obtained for A) unmodified product B) Acidic variant C) Basic 1 variant D) Basic 2 variant E) Basic 3 variant and F) Basic 4 variant by fitting Boltzmann function to the thermal denaturation data.

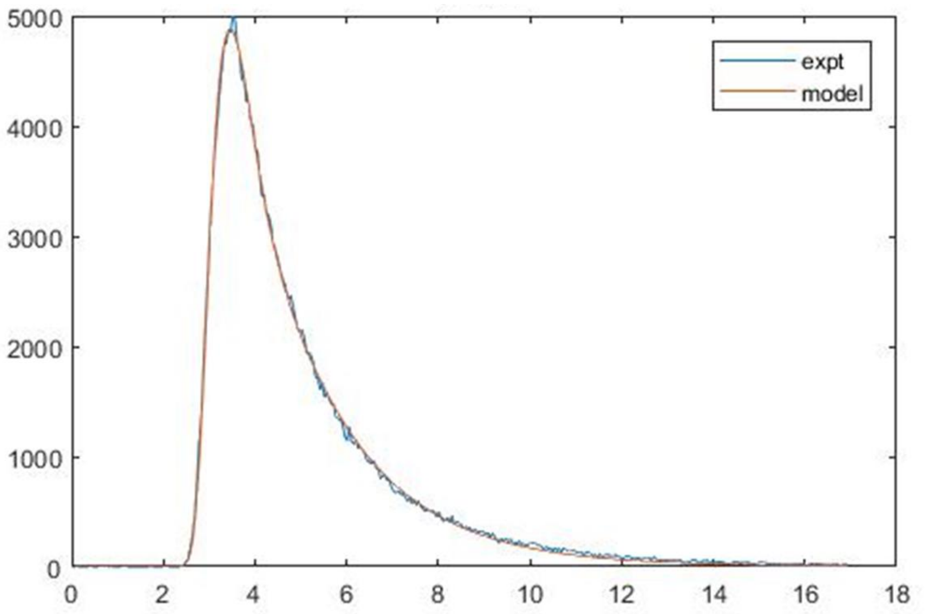

Decay Function is assumed to be sum of two exponentials

Alpha1*exp(-beta1*t) + alpha2*exp(-beta2*t)

Supplementary Figure 4: Determination of average fluorescence lifetime distribution of bevacizumab and its charge variants using time-correlated single photon counting spectroscopy. The samples were excited with a wavelength of 280nm using a light emitting diode and the decay profiles of the were monitored at 337nm at the magic angle (54.7^{o}C). The fluorescence lifetimes of the samples decayed following two stages: one in picosecond timescale and the other in the nanosecond timescale. Illustration of a typical decays profile of bevacizumab approximated as a sum of two exponentials using Marquardt least square minimization and fitting a four parameter fit to the decay profiles.

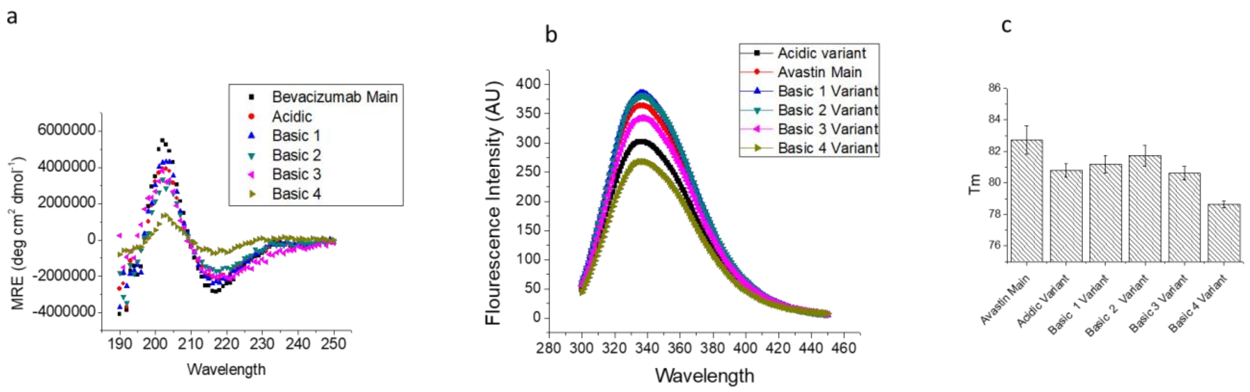

Supplementary Figure 5: (a) Overlay of the far-UV CD spectra of bevacizumab and its charge variants at a concentration of 0.2mg/ml. A mean minimum of 218nm were observed for all the variants suggesting a β-rich composition of the secondary structure (b) Fluorescence spectra of bevacizumab and its charge variants isolated from the semi-preparative cation exchange chromatography. Intrinsic fluorescence was monitored at 295nm. The emission spectra were recorded between 300-400 nm.

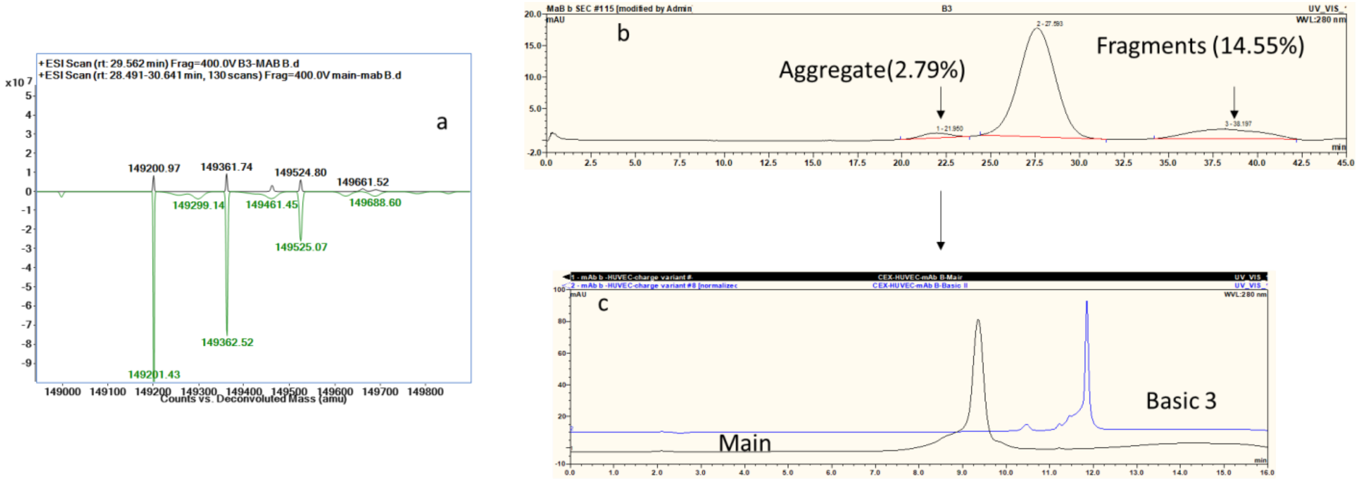

Supplementary Figure 6: (a) Mirror plot of deconvoluted mass spectrum of basic 3 variant and bevacizumab main product. No difference in the experimental and theoretical mass were noted. (b) Size exclusion chromatography trace of the basic 3 variant showing present of 3% aggregates and 14.5% fragments. (c) CEX chromatogram showing that shift in retention time (RT) of the basic 3 variant to that of bevacizumab main product RT after removal of the fragments from the sample.

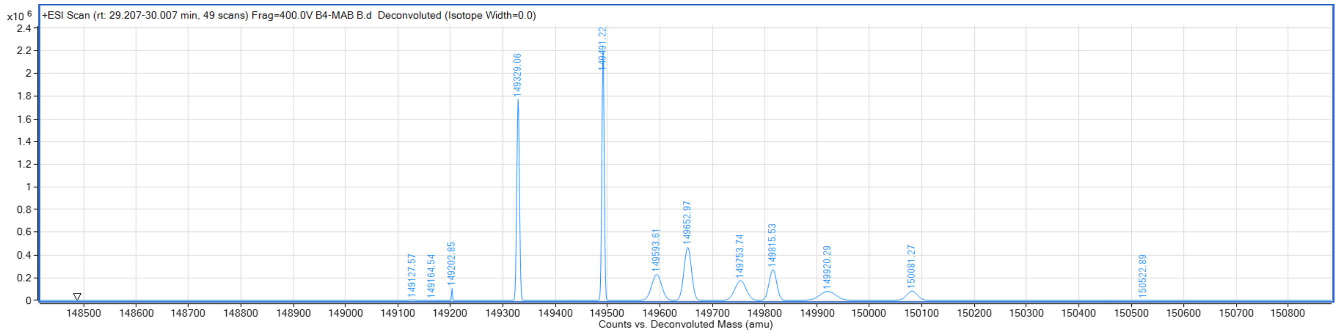

Supplementary Figure 7: Deconvoluted MS spectrum showing a shift of +32 Da mass from the expected intact mass of basic 4 variant. The observed charge heterogeneity is attributed to oxidation.

Supplementary Table 1: Listing the average fluorescence lifetimes, pre-exponential factors and fractional intensities of time resolved fluorescence decay for bevacizumab and its charge variants.

**Sample**	**Avg Fl. Lifetime**	**α1**	**α2**	**β1**	**β2**
Acidic	0.72	1.22	0.41	1.29	2.04
Main	0.73	1.37	0.33	1.32	1.98
B1	0.71	1.27	0.35	1.34	2.02
B2	0.74	1.20	0.46	1.24	2.12
B3	0.77	1.37	0.38	1.22	2.12
B4	0.73	1.12	0.48	1.25	2.17
